# Assessing the difference of tolerance and phytoremediation potential in mercury contaminated soil of a non-food energy crop, *Helianthus tuberosus* L. (Jerusalem artichoke)

**DOI:** 10.7717/peerj.4325

**Published:** 2018-02-01

**Authors:** Shiqi Lv, Bin Yang, Yixuan Kou, Jun Zeng, Ruixiong Wang, Yumeng Xiao, Fencan Li, Ying Lu, Yuwen Mu, Changming Zhao

**Affiliations:** State Key Laboratory of Grassland Agro-Ecosystems, School of Life Sciences, Lanzhou University, Lanzhou, Gansu, China

**Keywords:** Mercury, Non-food energy crop, Phytoremediation, Jerusalem artichoke

## Abstract

This study was conducted to evaluate the effects of mercury stress on growth, photosynthesis and mercury accumulation in different cultivars of a non-food energy crop, Jerusalem artichoke, and to screen appropriate cultivars for their efficacy in the phytoremediation of mercury (Hg^2+^) contaminated soil. Cultivars LZJ033 (high above-ground biomass and nutrient content, and strongly sexual reproduction) and LZJ119 (a long period of vegetative growth) exhibited more tolerance to mercury stress than LZJ047 (the highest tuber yield and total sugar content). The lines LZJ119 and LZJ047 showed delays in emergence time of about four weeks, and LZJ047 exhibited the highest mortality rate, 85.19%, under treatment with 10 mg kg^-1^ mercury. The MDA (malondialdehyde) content increased whereas and the *P*_*n*_ (net photosynthetic rate), *F*_*v*_∕*F*_*m*_ (the maximum quantum yield of PSII photochemistry) and chlorophyll content decreased in response to mercury stress. The stem diameter, stem biomass and photosynthetic rate of Jerusalem artichoke showed some modest increases in response to mercury stress and exhibited hormesis at least 1 mg kg^-1^ mercury treatment. Overall, LZJ119 produced more biomass under mercury stress, whereas LZJ033 exhibited a greater capacity for mercury bioaccumulation. Accordingly, LZJ119 may be a good candidate cultivar for use in cases of moderate—low mercury contamination, whereas LZJ033 may be a better candidate under conditions of high mercury contamination. When Jerusalem artichoke was cultivated in mercury contaminated soil, it not only removed the mercury from soil but also produced large amounts of tubers and shoots which could be used as feedstock for the production of bioethanol.

## Introduction

Heavy metal pollution is one of the most important ecological problems worldwide because significant amounts of these elements are released into the environment via natural and anthropogenic activities (Meng, Chen & Yang, 2011). Excessive levels of heavy metals (especially mercury) not only exert significantly detrimental effects on plant growth and crop yield, but also represent a threat to human health due to their bioaccumulation and biomagnification in food chains (Patra & Sharma, 2000; Zahir et al., 2005; Yadav, 2010). Mercury, which is one of the most toxic heavy metals commonly found in the global environment, has been widely used in seed disinfectants, fertilizers and pesticides. Natural mercury exists both in inorganic forms (e.g., Hg^0^, Hg^+^, Hg^2+^) and as organic compounds (e.g., methyl-Hg); the inorganic forms, especially Hg^2+^, are the predominant form in soils, from which they are readily absorbed by plants (Patra & Sharma, 2000; Gao et al., 2010; Chen & Yang, 2012).

In the 1950s, the problem of environmental pollution by mercury attracted much attention across the world because of the Minamata Bay tragedy (Zahir et al., 2005). As economies develop, mercury is being used more widely, and more and more refuse contains mercury which can be released into the environment. It has been estimated that in 2,000 the global average mercury level in arable lands was 39 kg km^−2^ (Chen & Yang, 2012). As mercury is a toxic metal, many studies have shown that, especially in the form of Hg^2+^, it readily accumulates in plant roots, though there is a barrier to translocation from roots to shoots, and that it has high phytotoxicity compared with other heavy metals (Munzuroglu & Geckil, 2002; Gautam et al., 2010). The phytotoxic effects of mercury include the following: reduction in plant growth and yield production, alterations in nutrient uptake and homeostasis, genotoxicity on genomic DNA molecule, elevated amounts of reactive oxygen species and membrane lipid peroxidation, changes in the integrity of bio-membranes, inhibition of plant photosynthesis, and perturbation of almost any function in which critical or non-protected proteins are involved due to mercury’s high affinity for sulphydryl groups (Patra & Sharma, 2000; Ali, Chun & Lee, 2002; Patra et al., 2004; Israr et al., 2006; Eibaz et al., 2010; Malar et al., 2015a; Malar et al., 2015b; Wang et al., 2015a).

As awareness of heavy metal pollution increases, many technologies are being developed to remediate land or water contaminated with heavy metals. Phytoremediation is a technology that uses plants to degrade, contain, or immobilize contaminants from soil and water, and it is regarded as the most promising approach for the remediation of heavy metal contaminated soil due to its environmental friendliness and the fact that it is potentially cost-effective compared with other methods (Tangahu et al., 2011). Hyperaccumulators are at the core of phytoremediation; they are plants that have a greatly enhanced rate of heavy metal uptake, more rapid root to shoot translocation and a greater ability to detoxify and sequester heavy metals in the leaves without suffering phytotoxic effects (Rascio & Izzo, 2011). Although to date about 450 angiosperm species have been identified as hyperaccumulators of heavy metals (e.g., Cd, Ni, Pb, Zn), no naturally-occurring plant species has yet been identified as a mercury hyperaccumulator (Su et al., 2008; Rascio & Izzo, 2011). There are many forms of phytoremediation, such as phytoextraction, phytostabilisation and so on. In the phytoextraction process, some specific plant species could absorb and hyperaccumulate contaminants in harvestable root and shoot tissue from the growth substrate, and in the phytostabilisation process the root plant excudates stabilize, demobilize and bind the contaminants in the soil matrix (Tangahu et al., 2011). Although phytoextraction is a good form of phytoremediation, it is difficult to find an appropriate plant for phytoextraction. It is less reported that the plants can be appled in phytoremediation with the form of phytoextraction in real; although Su et al. (2008) had reported Chinese brake fern was a potential candidate for mercury phytoextraction in experiments, Chinese brake fern has less biomass, and there is a lot of limitations in actual application.

*Helianthus tuberosus* L. also called Jerusalem artichoke, topinambur or sunchoke, is an annual herb plant which belongs to the Compositae and is a member of the sunflower genus; it originated in North America, and has been widely cultivated around the world as a feedstock for the production of bioethanol (Kou et al., 2013; Long et al., 2016). Jerusalem artichoke can produce large amounts of biomass, and it grows well on marginal land with little input of fertilizer and water; thus, it does not compete with food crops for arable land and therefore does not have negative effects on the food security of a country in which it is grown as a non-food energy crop. Tubers of Jerusalem artichoke are rich in inulin (70–90% of the dry weight), an oligosaccharide which is easily converted to biofuels (e.g., ethanol, butanol), thus Jerusalem artichoke is considered to be a good candidate for use as a non-food energy crop (Liu et al., 2012; Kou et al., 2013; Wang et al., 2015b; Long et al., 2016).

Information about the phytotoxic effects of mercury and mercury accumulation in Jerusalem artichoke is scanty. The aim of this study was to assess the phytotoxicity of mercury and its accumulation in Jerusalem artichoke from different groups grown in mercury-contaminated soil, and to carry out a screen in order to identify appropriate cultivars for the phytoremediation of mercury-contaminated soil.

## Materials and Methods

### Plant material and growth condition

Through previous analysis of the germplasm diversity of *Helianthus tuberosus* L. collected from East Asia and Europe, revealed by AFLP (Amplified Fragment Length Polymorphism) marker and phenotypic traits, three major genetic groups were identified: group I, which exhibited high above-ground biomass and nutrient content, and strongly sexual reproduction; group II, with a long period of vegetative growth; and group III, which had the highest tuber yield and total sugar content (Kou et al., 2013). We selected three representative cultivars (LZJ033, LZJ119 and LZJ047) representing, respectively, each of these three groups, as experimental materials.

This experiment was conducted in a rain-shelter at the Yuzhong experimental station of Lanzhou University, Gansu, China (35°56′N, 104°09′E, 1,750 m a.s.l.) in 2013. Five mercury treatments (control (CK), 0.15, 1, 5, 10 mg kg^−1^Hg^2+^(in the form of HgCl_2_)), with ten replicates for each, were set up and arranged in a completely randomized design. Topsoil (0–20 cm) from uncontaminated arable land (whose soil properties were as follows: soil pH 8.14; organic matter content 1.26%; available nitrogen 91.9 mg kg^−1^; available phosphorus 23 mg kg^−1^; available potassium 168.5 mg kg^−1^; total mercury content 0.036 mg kg^−1^) was put into plastic pots (35 cm in diameter and 30 cm in height), using about 15 kg (dry weight) per pot. The soil was then saturated with solution containing the required amount of HgCl_2_. One week later, the soil in every pot was thoroughly mixed and then three tubers, selected as having 1–2 buds, weighing about 15 g, and exhibiting no decay, were planted in each pot on April 10. All pots were watered every week according to soil water content in order to prevent drought stress.

### Measurements of agronomic traits

The germination rate was monitored every two weeks from the time of sowing until the 8th week, and the mortality rate was determined in the 20th week. Plant height, stem diameter and internode length were measured at maturity using, respectively, a tape measure, a vernier caliper and a portable leaf area system (LI-3000; Li-COR, Lincoln, NE, USA).

The stem and leaf tissue were cut off from the base of each plant on October 1 and washed with tap water followed by deionized water in order to remove any mercury adhering to the surface; meanwhile, the roots and tubers were dug up and vigorously washed with tap water and followed by deionized water and ultrasound bath in order to remove any soil particles and mercury adhering to the surface, and then the tubers were cut into sections about 0.5 cm thick using a knife. All plant materials were then dried at room temperature until constant weights were attained so as to avoid mercury loss.

### Determination of mercury content

The samples of plant material were ground to powder (particle size <0.15 mm) after weighing, then sealed into plastic bags for mercury analysis. Dried powder samples from the leaf, stem, tuber and root of the Jerusalem artichoke were digested with concentrated HNO_3_ and H_2_O_2_ in microwave about two hours and the resulting solutions were analyzed for mercury content using cold vapor atomic absorption spectrometry according to the method of Chen et al. (2009).

The bioaccumulation factor (BF), translocation factor (TF) and amounts of metal in the plants, which were used to evaluate the potential for phytoremediation of Jerusalem artichoke, were calculated from the following equations:

\begin{eqnarray*}& & \mathrm{BF}= \frac{\text{Metal concentration in plant tissue}}{\text{Metal concentration in soil}} \end{eqnarray*}

\begin{eqnarray*}& & \mathrm{TF}= \frac{\text{Metal concentration in plant tissue}}{\text{Metal concentration in root}} \end{eqnarray*}

Amounts of metal in plant tissue = Metal concentration in plant tissue × tissue biomass.

### Lipid peroxidation assay

The extent of leaf lipid peroxidation was determined by measuring the amount of malondialdehyde (MDA), an end-product of lipid peroxidation, by reaction with TBA (thiobarbituric acid) according to the method of Dhindsa, Dhindsa & Thorpe (1981), and the content of MDA was expressed as nmol g^−1^ fresh weight (FW).

### Analysis of photosynthetic characteristics

Leaf chlorophyll content was determined as follows: each leaf was homogenized in 80% ice-cold acetone in the dark and then centrifuged at 10,000 rpm for 10 min at 4 °C; the chlorophyll content of the supernatant was estimated using Arnon’s method (Arnon, 1949) and the chlorophyll content was expressed as mg g^−1^ FW.

The net photosynthetic rate (*P*_*n*_) of Jerusalem artichoke plants was measured in leaves (the sixth leaf from the top of the stem) using an Li-6400 portable photosynthesis system (Li-COR, Lincoln, NE, USA) with an Li-6400-02B LED light source in the middle of July. The light intensity, temperature, flow rate of the sample cell were kept as follows: 1,200 µmol m^−2^ s^−1^, 28 °C, 300 µmol s^−1^, and the CO_2_ concentration of reference cell was kept about 380 µmol mol^−1^ through a buffer bottle. All the measurements were carried out from 9:00 to 12:00 in the fine weather.

Maximum quantum yield of primary photochemistry was measured with a portable fluorimeter (Handy PEA, Hansatech, UK). The leaves were dark-adapted about 30 min before taking measurements, then exposed to red light supplied by an LED at a wavelength of 650 nm with an excitation irradiance at a density of 3,000 µmol m^−2^ s^−1^ and a duration of 800 ms, according to the methods of Wang et al. (2014). The minimal (*F*_0_) and maximal (*F*_*m*_) fluorescence yield parameters were obtained and the maximum quantum yield of PSII photochemistry was then calculated as the ratio *F*_*v*_∕*F*_*m*_ = (*F*_*m*_ − *F*_0_)∕*F*_*m*_.

### Statistical analysis

All treatments were arranged in a completely randomized design with ten replicates per treatment. Data were processed with Microsoft Excel 2007 and the SPSS 16.0 statistical software package. One-way ANOVA and post hoc least significant difference (LSD) tests were used to compare the agronomic and physiological parameters of different varieties under mercury treatment, two-way ANOVA (general linear model) was carried out to assess the effects of mercury treatments, cultivars and their interactions on the variables examined, before these comparisons the data were tested for homogeneity.

## Results

### Agronomic traits

Generally the agronomic traits of all cultivars changed in the same way when they were subjected to the different mercury treatments, and the mercury treatment, cultivar and the interaction between them had significant effects on plant height, internode length and leaf area (Table S1). As Table 1 shows, the final germination rate under mercury treatment ranged from 90% to 100%. Although the germination rate was thus little affected by mercury treatment, the seedling emergence time was delayed by this treatment. The emergence of LZJ047 and LZJ119 was delayed by about four weeks in the 10 mg kg^−1^ mercury treatment, though LZJ033 was less affected. We also observed some plant death at higher mercury concentrations, but there were differences among the three cultivars (Table 1). The mercury treatment killed the plants only at 10 mg kg^−1^ and the mortality rate was only 25.93% and 21.43% for, respectively, LZJ119 and LZJ033, but the mortality rate of LZJ047 was 85.19% under the 10 mg kg^−1^ mercury treatment, and mercury treatment at 5 mg kg^−1^ killed some LZJ047 plants; the mortality rate was 13.33%.

**Table 1:** The effects of mercury stress on germination and mortality of cultivars LZJ047, LZJ119 and LZJ033.

Cultivar	Soil mercury concentration (mg kg^−1^)	Germination rate (%)				Mortality rate (%)
		2nd week	4th week	6th week	8th week	20th week
LZJ047	CK	53.33	100	100	100	0
	0.15	50	96.67	100	100	0
	1	43.33	96.67	100	100	0
	5	20	66.67	96.67	100	13.33
	10	10	53.33	76.67	90	85.19
LZJ119	CK	60	100	100	100	0
	0.15	63.33	83.33	100	100	0
	1	40	86.67	100	100	0
	5	26.67	76.67	93.33	93.33	0
	10	16.67	70	83.33	90	25.93
LZJ033	CK	56.67	96.67	100	100	0
	0.15	60	100	100	100	0
	1	46.67	96.67	100	100	0
	5	23.33	96.67	100	100	0
	10	20	93.33	93.33	93.33	21.43

**Figure 1:**
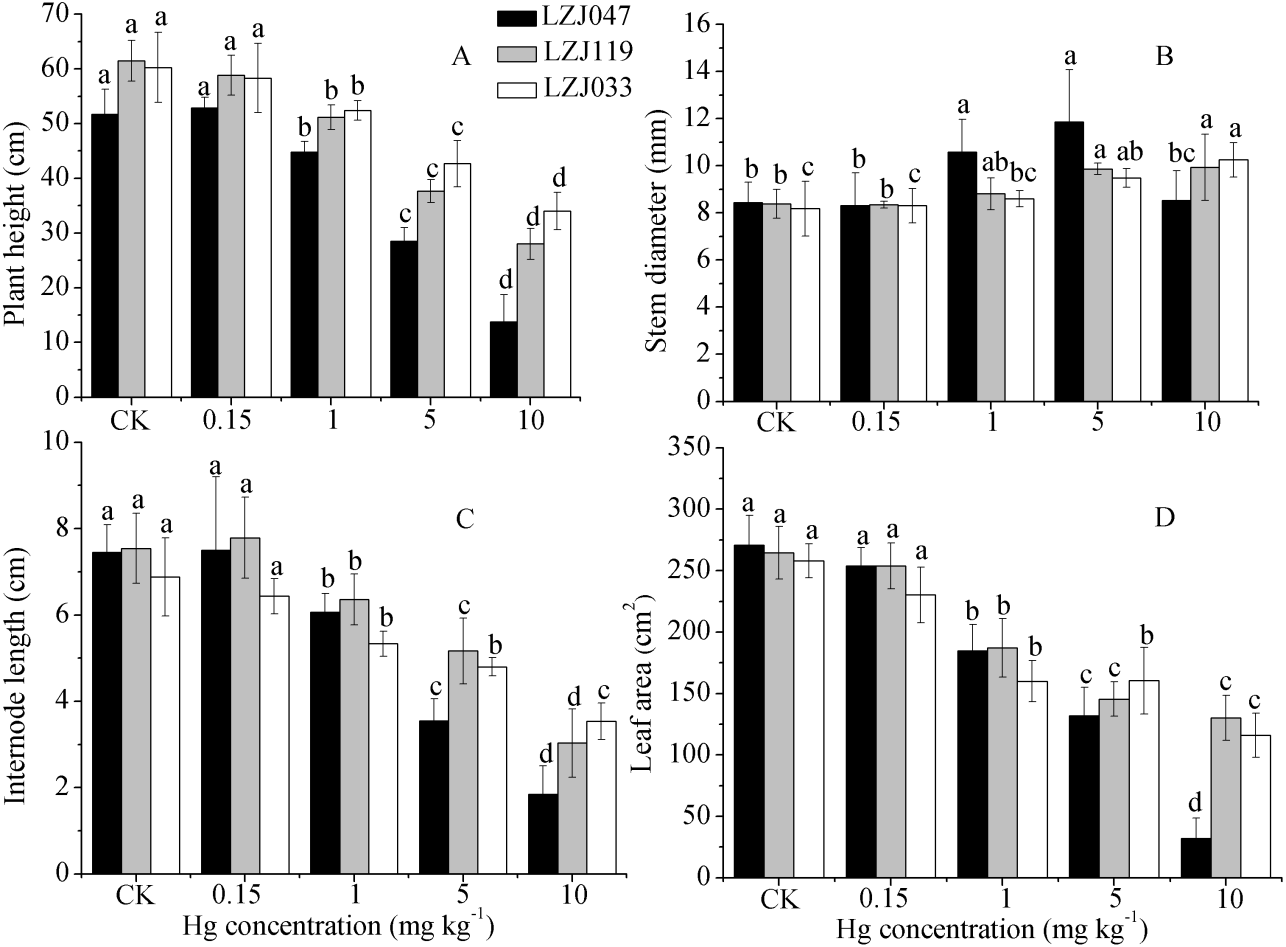
The effects of mercury stress on plant height (A), stem diameter (B), internode length (C) and leaf area (D) of LZJ047, LZJ119 and LZJ033 after six months of cultivation. Means and standard deviation (SD) values of at least three replicates are shown and the letters that follow the SD values denote statistically significant differences (*p* < 0.05) among the mercury treatments.

As shown in Fig. 1, a major inhibitory effect on all growth parameters except stem diameter was observed with increasing concentration of mercury in the medium; plant height, internode length and leaf area of LZJ047, LZJ119 and LZJ033 were significantly decreased by, respectively 13.24%, 18.66%, 31.74%; 16.80%, 22.53%, 29.25% and 13.03%, 22.53%, 38% of control values in the 1 mg kg^−1^ mercury treatment and the values were reduced by 73.39%, 75.17%, 88.12%; 54.47%, 59.81%, 57.06% and 43.57%, 48.69%, 55.06% respectively compared with control values under 10 mg kg^−1^ mercury treatment.

Although the plant height, internode length and leaf area were reduced, stem diameter exhibited some increase in response to mercury treatment. As shown in Fig. 1, the stem diameter of LZJ119 and LZJ033 significantly increased, by 17.64% and 18.36% respectively, in the 5 mg kg^−1^ mercury treatment, and the increases were, respectively, 18.36% and 25.31% in the 10 mg kg^−1^ mercury treatment compared with the control. The stem diameter of LZJ047 significantly increased, by 25.36% and 40.52% in the 1 mg kg^−1^ and 5 mg kg^−1^ mercury treatment respectively, but it only increased by 1.07% in the 10 mg kg^−1^ mercury treatment and the increase was not significant.

### Lipid peroxidation and photosynthetic characteristics

**Figure 2:**
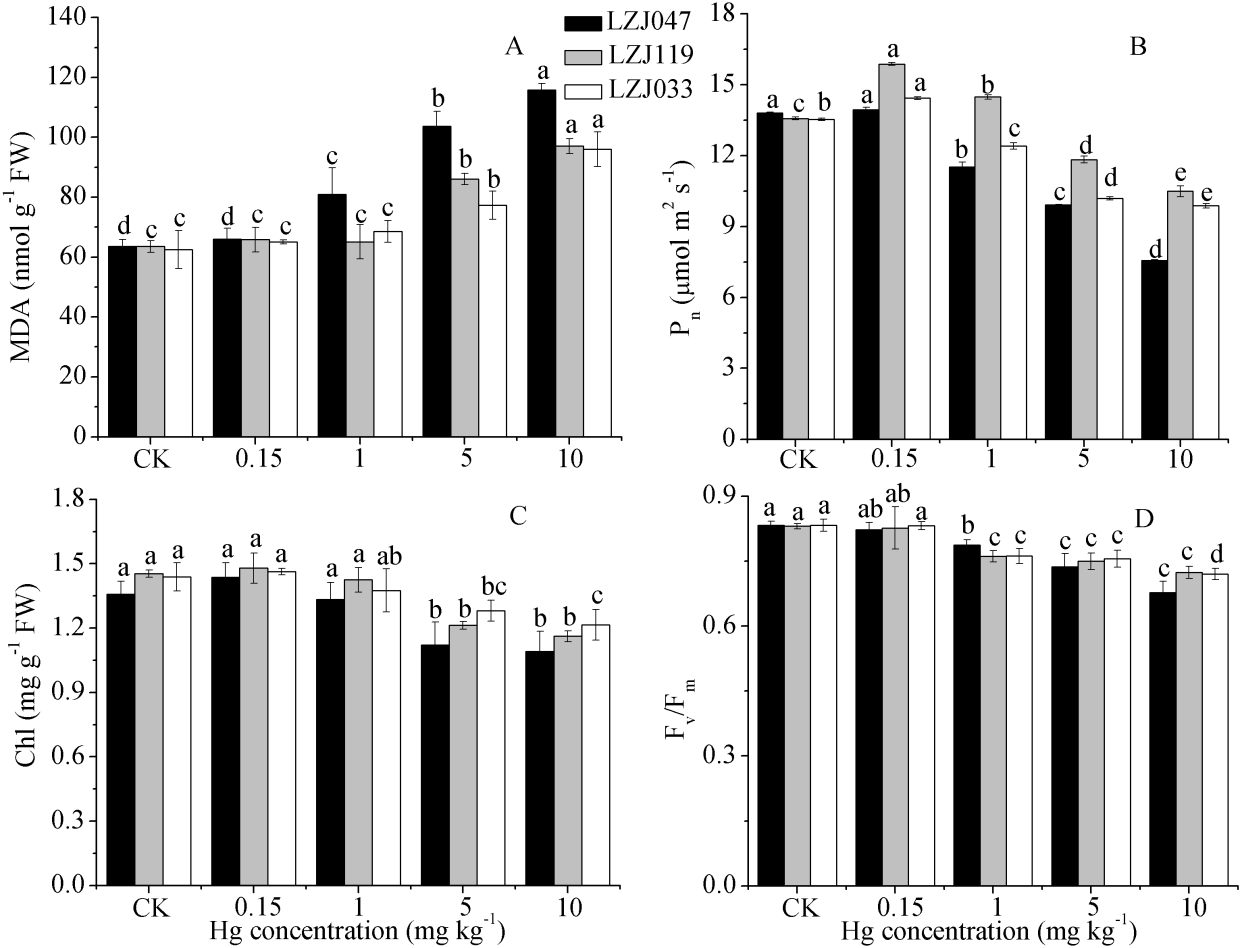
The effects of mercury stress on MDA content (A), *P*_*n*_ (B), chlorophyll content (C) and *F*_*v*_∕*F*_*m*_ (D) of LZJ047, LZJ119 and LZJ033 after two months of cultivation. Means and standard deviation (SD) of three replicates are shown and the letters that follow the SD values denote statistically significant differences (*p* < 0.05) among the mercury treatments.

The effects of mercury on lipid peroxidation and photosynthetic traits are shown in Fig. 2; the MDA content, which is a good indicator of lipid peroxidation, increased concurrently with an increase in external mercury concentration. The MDA content significantly increased, by 27.54%, in LZJ047 exposed to 1 mg kg^−1^ mercury, and by 35.44% and 23.63% in LZJ119 and LZJ033 in a mercury concentration of 5 mg kg^−1^ compared with control plants; the increases for the three cultivars reached 82.4%, 52.83% and 53.67% when they were exposed to 10 mg kg^−1^ mercury. In general LZJ047 had a high MDA content, which indicated that LZJ047 may be especially susceptible to mercury stress.

As shown in Fig. 2, the net photosynthetic rate increased by 16.86% and 6.57% under a 0.15 mg kg^−1^ mercury concentration, representing significant changes, in, respectively, LZJ119 and LZJ047, and it then decreased with a further increase of external mercury concentration. The P _n_ value significantly decreased, by 12.89%, in LZJ119 exposed to 5 mg kg^−1^ mercury, and by 16.52% and 8.35% in LZJ047 and LZJ033 in 1 mg kg^−1^mercury; the decline in response to 10 mg kg^−1^ mercury was 45.14%, 22.75%, 27.03% in, respectively, LZJ047, LZJ119, LZJ033 compared with the control .

The chlorophyll content and *F*_*v*_∕*F*_*m*_ decreased as external mercury concentration increased (Fig. 2); chlorophyll content and *F*_*v*_∕*F*_*m*_ significantly decreased under 5 mg kg^−1^ and 1 mg kg^−1^ mercury concentrations and the respective reductions were 19.85%, 20%, 15.28% and 18.73%, 12.88% 13.57% when 10 mg kg^−1^ mercury was applied to, respectively, LZJ047, LZJ119, LZJ033.

### Plant biomass

**Figure 3:**
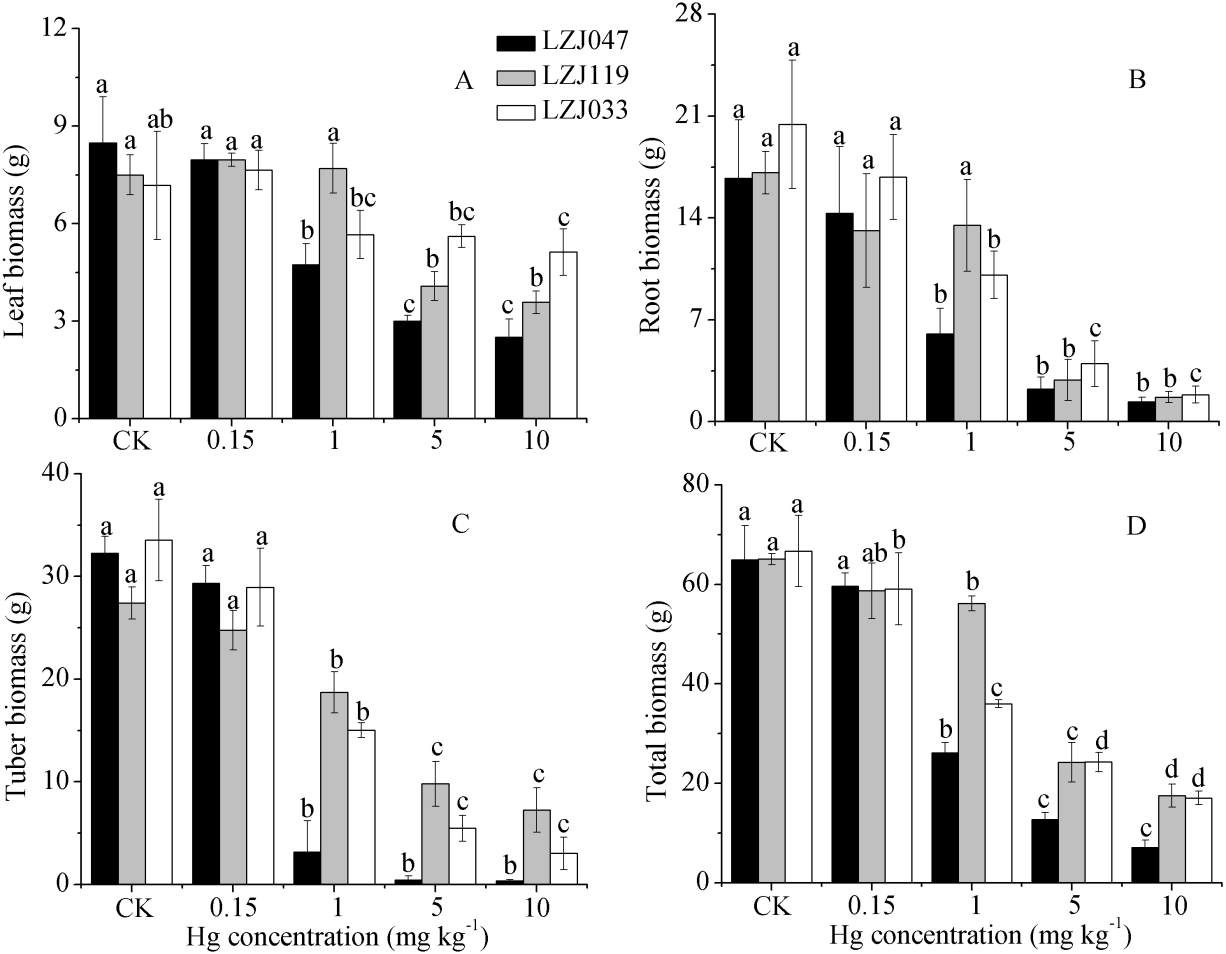
The effects of mercury stress on leaf biomass (A), root biomass (B), tuber biomass (C) and total biomass (D) of LZJ047, LZJ119 and LZJ033 after six months of cultivation. Means and standard deviation (SD) values of three replicates are shown and the letters that follow the SD values denote statistically significant differences (*p* < 0.05) among the mercury treatments.

Mercury exposure induced a substantial decrease in dry weights in Jerusalem artichoke. As shown in Fig. 3, the leaf, root, tuber and total biomass of LZJ047 significantly decreased, by respectively 44.22%, 63.90%, 90.20% and 59.83% compared with the control, in response to 1 mg kg^−1^ mercury, and the declines were 70.40%, 91.87%, 98.88%, 89.10% at a mercury concentration of 10 mg kg^−1^. In LZJ119, tuber and total biomass significantly decreased, by 31.75 and 13.69% respectively, in response to 1 mg kg^−1^ mercury; the leaf and root biomass were observed to decrease significantly (by 45.6% and 83.28%) in 5 mg kg^−1^ mercury, and the reductions in these four biomass parameters reached, respectively, 64.23%, 73.08%, 53.27% and 90.18% compared with the control in the 10 mg kg^−1^ mercury treatment. Although the root, tuber and total biomass of LZJ033 significantly decreased, by 50.66%, 55.28%, 46.03% respectively, in response to 1 mg kg^−1^ mercury, the leaf biomass decreased significantly, by 28.55%, only when 10 mg kg^−1^ mercury was present, and the declines were, respectively, 90.94%, 91% and 74.43% compared with the control in response to 10 mg kg^−1^ mercury.

**Figure 4:**
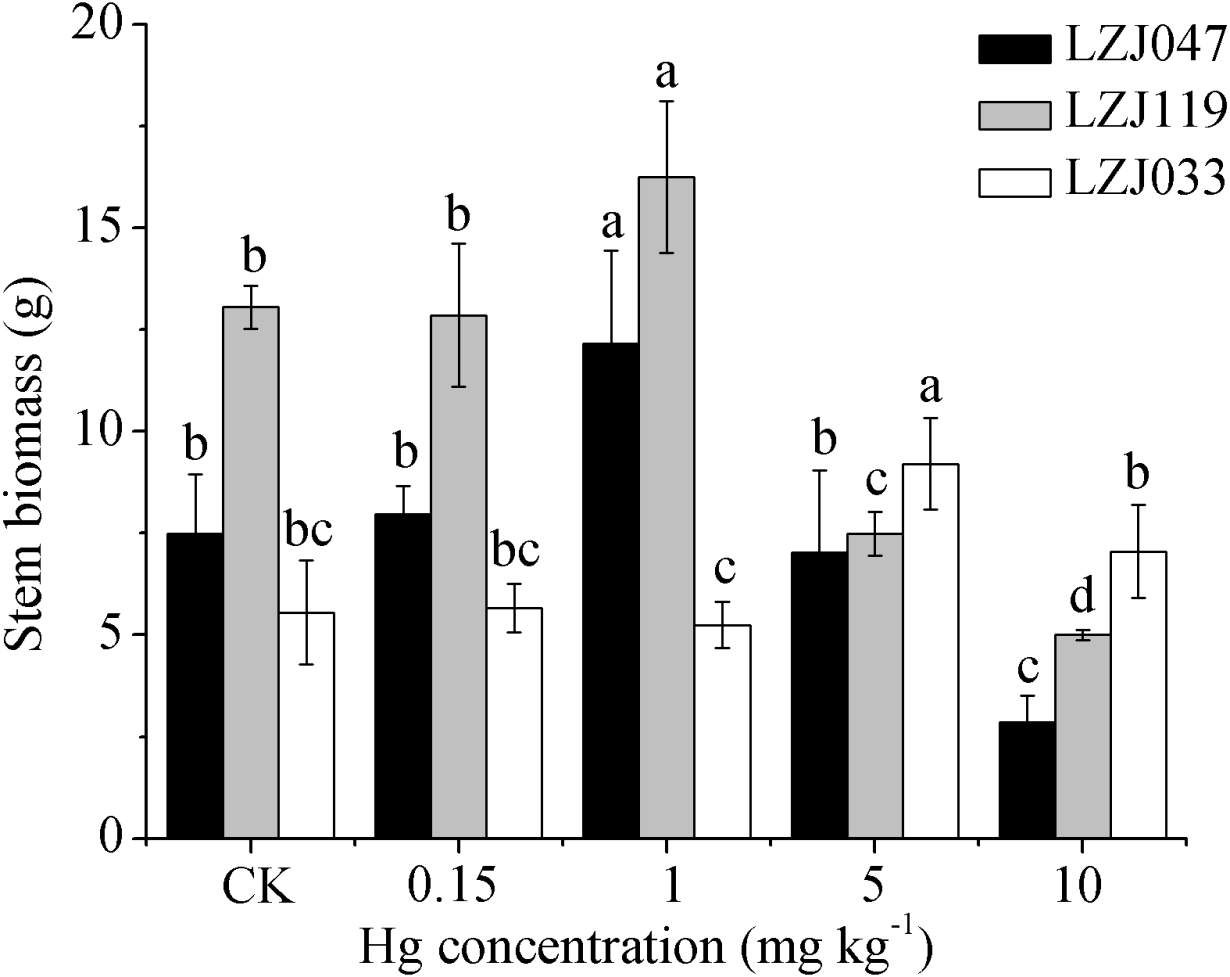
The effects of mercury stress on stem biomass of LZJ047, LZJ119 and LZJ033. Means and standard deviation (SD) values of three replicates are shown and the letters that follow the SD values denote statistically significant differences (*p* < 0.05) among the mercury treatments.

Unlike the decline in the biomass of other organs, the stem biomass was observed to increase to some extent when Jerusalem artichoke was subjected to mercury treatment (Fig. 4). The stem biomass of LZJ047 was significantly increased, by 62.35%, in response to 1 mg kg^−1^ mercury; it then decreased and was at the significantly lower level of 61.95% of the control value at a mercury concentration of 10 mg kg^−1^; and the stem biomass of LZJ119 underwent changes similar to those of LZJ047. The stem biomass of LZJ033 significantly increased (by 65.77%) in response to 5 mg kg^−1^ mercury; no significant change was observed at other mercury concentrations.

### Plant mercury contents

Mercury treatment, cultivar and the interaction between them had significant effects on the mercury concentration in plant organs, with the exception of tubers (Table S1 ); the concentration of mercury in the tissues of the three Jerusalem artichoke cultivars increased with an increase of external mercury concentration, and the mercury concentration of roots was higher than that of other organs (Table 2). As shown in Table 3, the mercury concentrations of leaf, stem, root and tuber in LZJ047 were, respectively, 10.89. 11.67, 93.98 and 16.06 times greater than those of control plants, and similar changes were observed in LZJ119; the mercury concentration of the four tissues was greater in LZJ033 than in the other two varieties, and the mercury concentration in roots of LZJ033 reached a maximum level of 1,269.8 µg g^−1^, which was, respectively, 4.13 times and 2.7 times greater than the maximum values for LZJ047 and LZJ119.

**Table 2:** The effects of mercury stress on mercury concentration in organs of the cultivars LZJ047, LZJ119 and LZJ033. Means and standard deviation (SD) values of three replicates are shown and the letters that follow the SD values denote statistically significant differences (*p* < 0.05) among the mercury treatment.

Cultivar	Soil mercury concentration (mg kg^−1^)	Leaf mercury concentration (µg g^−1^)	Stem mercury concentration (µg g^−1^)	Root mercury concentration (µg g^−1^)	Tuber mercury concentration (µg g^−1^)
LZJ047	CK	2.57 ± 0.10d	2.44 ± 0.01d	3.27 ± 0.90d	2.36 ± 0.12b
	0.15	2.66 ± 0.08d	2.47 ± 0.03d	5.49 ± 0.58d	2.74 ± 0.08b
	1	4.03 ± 0.45c	3.18 ± 0.39c	41.21 ± 14.78c	7.42 ± 4.65b
	5	25.43 ± 0.39b	25.03 ± 0.40b	148.92 ± 2.08b	33.93 ± 0.72a
	10	28 ± 0.5a	28.47 ± 0.64a	307.33 ± 9.17a	37.9 ± 7.85a
LZJ119	CK	2.85 ± 0.39d	3.45 ± 0.99b	4.00 ± 0.63d	3.48 ± 0.13c
	0.15	4.05 ± 0.12cd	3.96 ± 0.12b	7.72 ± 0.34cd	4.21 ± 0.18bc
	1	4.87 ± 0.33c	4.65 ± 0.53b	39.22 ± 9.93c	10.52 ± 0.13b
	5	26.55 ± 1.48	29.07 ± 0.93a	119.47 ± 22.44b	31.17 ± 6.74a
	10	33.4 ± 1.04a	29.93 ± 2.05a	469.75 ± 21.87a	37.17 ± 4.05a
LZJ033	CK	2.12 ± 0.09c	2.38 ± 0.42c	2.78 ± 0.31d	2.47 ± 0.28c
	0.15	2.34 ± 0.12c	2.29 ± 0.07c	6.42 ± 1.35d	2.96 ± 0.29c
	1	3.94 ± 0.61c	3.47 ± 0.65c	46.93 ± 5.78c	3.84 ± 3.8c
	5	28.7 ± 8.42b	20.3 ± 1.41b	257.43 ± 37.50b	35.3 ± 3.8b
	10	57.83 ± 1.68a	32.37 ± 0.15a	1269.8 ± 15.33a	39.5 ± 1.23a

**Table 3:** The effects of mercury stress on BF and TF of plant organs of LZJ047, LZJ119 and LZJ033. Means and standard deviation (SD) values of three replicates are shown and the letters that follow the SD values denote statistically significant differences (*p* < 0.05) among the mercury treatments.

Cultivar	Soil mercury concentration (mg kg^−1^)	BF of leaf	BF of stem	BF of root	BF of tuber	TF of leaf	TF of stem	TF of tuber
LZJ047	CK	71.48 ± 2.80a	67.69 ± 0.32a	90.83 ± 24.98a	65.65 ± 3.36a	0.82 ± 0.18a	0.78 ± 0.18a	0.76 ± 0.21a
	0.15	17.71 ± 0.52b	16.49 ± 0.20b	36.58 ± 3.86b	18.29 ± 0.54b	0.49 ± 0.06b	0.45 ± 0.04b	0.50 ± 0.04b
	1	4.03 ± 0.45c	3.18 ± 0.39de	41.21 ± 14.78b	7.42 ± 4.65c	0.10 ± 0.03c	0.08 ± 0.03c	0.21 ± 0.18c
	5	5.09 ± 0.08c	5.01 ± 0.08c	29.78 ± 0.42b	6.78 ± 0.14c	0.17 ± 0.002c	0.17 ± 0.0007c	0.23 ± 0.003c
	10	2.8 ± 0.05c	2.85 ± 0.06e	30.73 ± 0.92b	3.79 ± 0.78c	0.09 ± 0.002c	0.09 ± 0.002c	0.12 ± 0.02c
LZJ119	CK	79.17 ± 10.82a	95.74 ± 27.58a	111.02 ± 17.52a	96.76 ± 3.70a	0.73 ± 0.17a	0.89 ± 0.36a	0.88 ± 0.10a
	0.15	27.02 ± 0.80b	26.4 ± 0.82b	51.47 ± 2.26b	28.09 ± 1.21b	0.53 ± 0.01b	0.52 ± 0.002b	0.55 ± 0.001b
	1	4.87 ± 0.33c	4.65 ± 0.053c	39.22 ± 9.93bc	10.52 ± 0.13c	0.13 ± 0.04c	0.12 ± 0.02c	0.28 ± 0.06c
	5	5.31 ± 0.30c	5.81 ± 0.19c	23.89 ± 4.49c	6.23 ± 1.35d	0.25 ± 0.07c	0.25 ± 0.05bc	0.27 ± 0.06c
	10	3.34 ± 0.10c	2.99 ± 0.21c	46.98 ± 2.19b	3.72 ± 0.40d	0.07 ± 0.003c	0.06 ± 0.01c	0.08 ± 0.007d
LZJ033	CK	58.89 ± 2.37a	66.20 ± 11.79a	77.13 ± 8.52b	68.52 ± 7.76a	0.77 ± 0.12a	0.88 ± 0.26a	0.89 ± 0.04a
	0.15	15.62 ± 0.81b	15.31 ± 0.44b	42.82 ± 9.03c	19.72 ± 1.95b	0.38 ± 0.08b	0.37 ± 0.08b	0.48 ± 0.14b
	1	3.94 ± 0.61c	3.47 ± 0.66c	46.93 ± 5.78c	3.84 ± 0.50c	0.08 ± 0.02c	0.07 ± 0.02c	0.08 ± 0.003c
	5	5.74 ± 1.68c	4.06 ± 0.28c	51.49 ± 7.50c	7.06 ± 0.76c	0.11 ± 0.02c	0.08 ± 0.01c	0.14 ± 0.006c
	10	5.78 ± 0.17c	3.24 ± 0.02c	126.98 ± 1.53a	3.95 ± 0.12c	0.04 ± 0.001c	0.03 ± 0.0004c	0.03 ± 0.001c

Although the mercury treatment, cultivar and the interaction between them had significant effects on BF, only the mercury treatment had significant effects on TF (Table S1). The BF and TF of these Jerusalem artichokes both altered in response to mercury treatment, as shown in Table 3. The BF significantly decreased and its level was low (below 10 in all tissues except root) under medium-high mercury concentration conditions. The BF of roots was seen to increase at a mercury concentration of 10 mg kg^−1^ and it reached its highest level, 126.98, in LZJ033, where it was 4.13 and 2.7 times greater than in, respectively, LZJ047 and LZJ119. The BF of shoots and tubers of LZJ119 was higher than that of the other two cultivars under medium-low mercury stress. The TF responded similarly to BF in these Jerusalem artichoke cultivars, remaining at a low level in medium-high mercury concentrations; and the TF values in all three cultivars were less than 0.1 (except in the case of tubers in LZJ047) at 10 mg kg^−1^ mercury.

### Amount of mercury in plant

**Figure 5:**
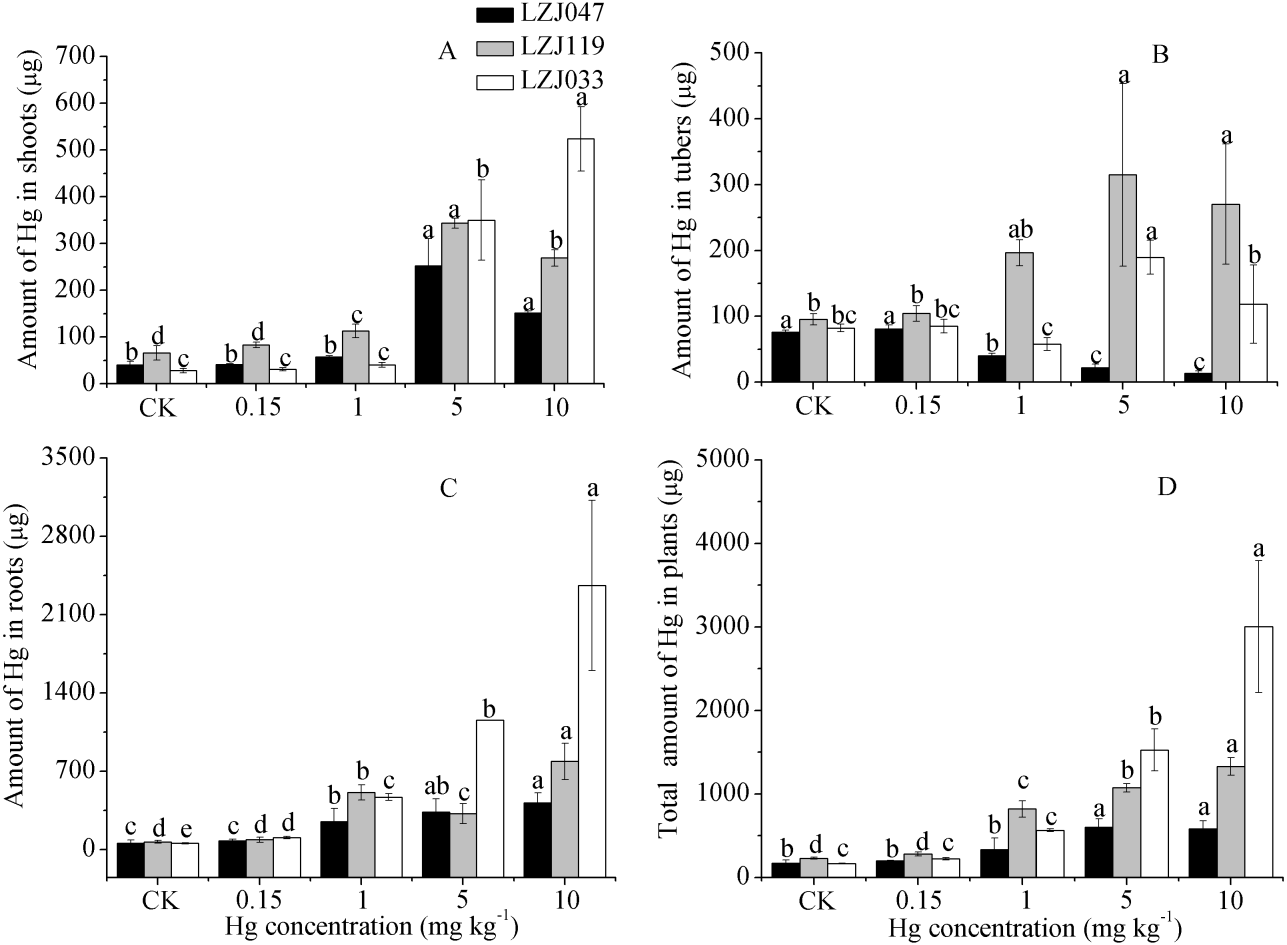
The effects of mercury stress on the amounts of mercury absorbed by plants in shoot (A), tuber (B), root (C) and total organs (D) of cultivars LZJ047, LZJ119 and LZJ033. Means and standard deviation (SD) values of three replicates are shown and the letters that follow the SD values denote statistically significant differences (*p* < 0.05) among the mercury treatment.

The amounts of mercury absorbed by plants were determined from the mercury concentrations and biomasses of the plants. As shown in Table S1, mercury treatment, cultivar and the interaction between them had significant effects on the amounts of mercury in the plants. As Fig. 5 shows, the mercury amounts absorbed increased with an increase in external mercury concentration, reaching maximum of 580.9, 1327.7 and 3,001.92 µg. The root had greater amounts of mercury than other organs at medium-high mercury concentrations, and the highest level, 2,359.6 µg in LZJ033, was attained under treatment with 10 mg kg^−1^ mercury. The maximum mercury content of tubers, 314.84 µg, was observed in LZJ119 at 5 mg kg^−1^ mercury, and the largest amount in shoots, 523.9 µg, was found in LZJ033 at 10 mg kg^−1^ mercury. The tuber and shoot are the main parts of Jerusalem artichoke to be harvested. The total mercury amounts of absorbed by tuber and shoot were reached to the highest level of 309.6, 658.5, 642.3 of LZJ119, LZJ119 and LZJ033 in 1 mg kg^−1^, 5 mg kg^−1^ and 10 mg kg^−1^ mercury treatment, respectively.

## Discussion

Mercury is not essential for plants, and it can have very toxic effects on plant growth (Patra & Sharma, 2000; Malar et al., 2015a; Malar et al., 2015b). In the present study, leaves progressively dried out from the tip at high mercury concentrations, until the plants eventually died. Mercury severely inhibited some growth of the three Jerusalem artichoke cultivars (only stem diameter and stem biomass were not reduced), delayed the germination of tubers, and increased the mortality rate, especially that of LZJ047. Previous study have indicated that mercury strongly suppresses growth in *Miscanthus,* maize, cucumber, tomato, alfalfa, field bean and other plant species (Hsu & Chou, 1992; Cho & Park, 2000; Cargnelutti et al., 2006; Rellán-Álvarez et al., 2006; Zhou, Wang & Yang, 2007; Muddarisna et al., 2013; D’Souza & Devaraj, 2013), and it significantly decreased the germination percentage of *Bowiea volubilis* and *Eucomis autumnalis* at a low concentration (0.5 mg l^−1^) (Street et al., 2007). Hormesis is a dose–response phenomenon which is characterized by low-dose stimulation and high-dose inhibition (Calabrese & Blain, 2009). In the past few decades there has been considerable interest in using phytoremediation to treat soil at contaminated sites, leading to numerous studies that have reported hormetic effects (Calabrese & Blain, 2009). The papers of Gardea-Torresdey et al. (2005) and Jia et al. (2013) showed that low concentrations of Cr and Cd have hermetic effects on some growth characteristics in *Salsola kali* and *Lonicera japonica*, such as root length, shoot length, root biomass, leaf biomass and chlorophyll content. The present study shows that the stem diameter, stem biomass and photosynthetic rate of Jerusalem artichoke undergo modest increases in response to mercury treatment, indicating the occurrence of hormesis in this species.

Mercury causes many biochemical and physiological changes in growing plants, including the generation of reactive oxygen species closely linked to oxidative damage in plant tissues, damage which is manifested as lipid peroxidation and loss of membrane integrity; the abundance of MDA, an important byproduct of lipid peroxidation, increases as oxidative damage occurs (Patra & Sharma, 2000; Chen & Yang, 2012). In the present study, the MDA content of the three Jerusalem artichoke cultivars increased with increasing mercury concentration. This has also been widely found in, for example, field bean, tomato, *Zea mays*, *Pteris vittata* and *Nephrolepis exaltata* under mercury treatment (Cho & Park, 2000; Rellán-Álvarez et al., 2006; Chen et al., 2009; D’Souza & Devaraj, 2013). Mercury affects photosynthesis, perturbing both light and dark reactions (Patra & Sharma, 2000). In the present study, the photosynthetic activity of Jerusalem artichoke was assessed by measuring the chlorophyll content, photosynthetic rate (*P*_*n*_) and maximum quantum yield of primary photochemistry (*F*_*v*_∕*F*_*m*_), all of which significantly decreased when the medium had a high mercury concentration. Numerous studies have shown similar changes; for example, Deng et al. (2013) reported that chlorophyll content and maximum quantum yield of primary photochemistry decreased with increasing mercury concentration, causing a decline in photosynthetic activity, in *Microsorum pteropus*. Yu et al. (2008) showed that a high mercury concentration significantly decreased chlorophyll content, maximum quantum yield of primary photochemistry and photosynthetic rate in tobacco. The ratio *F*_*v*_∕*F*_*m*_ is often used as a stress indicator, with values above 0.8 being characteristic of healthy plants (Dan, Krishnaraj & Saxena, 2000). In our study, the maximum quantum yield of primary photochemistry was affected only by mercury treatment, not by cultivar or the interaction between mercury treatment and cultivar (Table 3), and the *F*_*v*_∕*F*_*m*_ ratio was less than 0.8 when the mercury concentration was greater than 1 mg kg^−1^; since it was more sensitive to mercury than the other two photosynthetic parameters measured here, we conclude that it can be used as an appropriate stress indicator for Jerusalem artichoke exposed to mercury.

The results of the present study suggest that there is a continuous increase in the mercury content in the tissues of Jerusalem artichoke as the external mercury concentration increases; the mercury is mainly accumulated in the roots since there is a barrier limiting the transport of mercury to shoots and tubers. The Jerusalem artichoke is obviously not a mercury hyperaccumulator, and the phytostabilisation mmybe more suitable for jerusalem artichoke in our study. This is in agreement with findings in willow, tomato, Indian mustard and cucumber (Cho & Park, 2000; Wang & Maria, 2004; Cargnelutti et al., 2006; Shiyab et al., 2009). Although phytoextraction is a good form of phytoremediation, it is difficult to find an appropriate plant for phytoextraction. There is less reported that the plants can be appled in phytoremediation with the form of phytoextraction in real, even though Su et al. (2008) had reported Chinese brake fern was a potential candidate for mercury phytoextraction, but Chinese brake fern is less biomass, and there is a lot of limitations in actual application. phytostabilisation is still a good form of phytoremediation with high biomass and mercury contains espically in harvest organs. In order to achieve remediation of soil mercury contamination as rapidly as possible, it is important to maximize the amounts of mercury accumulated in the harvested organs. Tubers and shoots are the main organs that are harvested from Jerusalem artichoke, hence harvesting these organs at the mature stage represents the most effective approach to the phytoremediation of mercury-contaminated soil. The results of the current study suggested that LZJ119 and LZJ033 exhibit greater tolerance to mercury stress than LZJ047, and that whereas LZJ119 produces more biomass under mercury treatment, LZJ033 has a greater capacity for mercury bioaccumulation. Overall, the cultivar LZJ119 is a good candidate for use in conditions of moderate–low mercury contamination and LZJ033 may be more suitable under high mercury contamination. Cultivating appropriate cultivars of Jerusalem artichoke in metal-contaminated soil can not only result in phytoremediation of the contamination but also yield large amounts of tubers and shoots for use as feedstock in the production of bioethanol.

## Conclusion

Our results showed that the Jerusalem artichoke is obviously not a mercury hyperaccumulator, the phytostabilisation maybe more suitable for Jerusalem artichoke and there is variation in the tolerance of Jerusalem artichoke from different groups to mercury stress. The cultivars LZJ033 and LZJ119, which are members of group I and group II, respectively exhibited more tolerance to mercury than LZJ047 from group III. LZJ119 produced more biomass, whereas LZJ033 exhibited a greater capacity for mercury bioaccumulation in response to mercury treatment; thus, LZJ119 is a good candidate for cultivation under moderate-low mercury contamination, but LZJ033 may be preferable for use at high levels of mercury contamination. Although our study is far away from fields, there is still a good indication for applying Jerusalem artichoke in the restoration of contaminated soil in fields.

##  Supplemental Information

10.7717/peerj.4325/supp-1 Table S1 Summary of two-way ANOVA results for all parameters, with the exceptions of germination and mortality rate, examined in this experiment; the statistically significant level was considered to be *P* < 0.05

10.7717/peerj.4325/supp-2 Data S1 Raw data of Fig. 1

10.7717/peerj.4325/supp-3 Data S2 Raw data of Fig. 2

10.7717/peerj.4325/supp-4 Data S3 Raw data of Figs. 3 and 4

10.7717/peerj.4325/supp-5 Data S4 Raw data of Table 1

10.7717/peerj.4325/supp-6 Data S5 Raw data of Hg concentration

## Additional Information and Declarations

### Data Availability

The following information was supplied regarding data availability: The raw data is uploaded as a Supplemental File.
