# Supplementary material for: Assessing the difference of tolerance and phytoremediation potential in mercury contaminated soil of a non-food energy crop, Helianthus tuberosus L. (Jerusalem artichoke)
Source: PeerJ. 2018 Feb 1;6:e4325. doi: 10.7717/peerj.4325 (PMC5797682; doi:10.7717/peerj.4325)
Supplement: Table S1 [file peerj-06-4325-s001.docx]

Table S1: Summary of two-way ANOVA results for all parameters, with the exceptions of germination and mortality rate, examined in this experiment; the statistically significant level was considered to be P<0.05.

| Parameters | Hg treatment | cultivar | Hg × cultivar |
| --- | --- | --- | --- |
| Plant height | **P<0.01** | **P<0.01** | **P<0.01** |
| Stem diameter | **P<0.01** | P=0.17 | **P<0.01** |
| Internode length | **P<0.01** | **P<0.01** | **P<0.01** |
| Leaf area | **P<0.01** | **P<0.01** | **P<0.01** |
| MDA | **P<0.01** | **P<0.01** | **P<0.01** |
| Pn | **P<0.01** | **P<0.01** | **P<0.01** |
| Chl | **P<0.01** | **P<0.05** | P=0.61 |
| Fv/Fm | **P<0.01** | P=0.5 | P=0.15 |
| Leaf biomass | **P<0.01** | **P<0.01** | **P<0.01** |
| Stem biomass | **P<0.01** | **P<0.01** | **P<0.01** |
| Root biomass | **P<0.01** | **P<0.05** | P=0.17 |
| Tuber biomass | **P<0.01** | **P<0.01** | **P<0.01** |
| Total biomass | **P<0.01** | **P<0.01** | **P<0.01** |
| Mercury concentration in leaf | **P<0.01** | **P<0.01** | **P<0.01** |
| Mercury concentration in stem | **P<0.01** | **P<0.01** | **P<0.01** |
| Mercury concentration in root | **P<0.01** | **P<0.01** | **P<0.01** |
| Mercury concentration in tuber | **P<0.01** | P=0.90 | P=0.33 |
| BF of leaf | **P<0.01** | **P<0.01** | **P<0.01** |
| BF of stem | **P<0.01** | **P=0.01** | **P=0.03** |
| BF of root | **P<0.01** | **P<0.01** | **P<0.01** |
| BF of tuber | **P<0.01** | **P<0.01** | **P<0.01** |
| TF of leaf | **P<0.01** | P=0.08 | P=0.58 |
| TF of stem | **P<0.01** | P=0.23 | P=0.90 |
| TF of tuber | **P<0.01** | P=0.05 | P=0.33 |
| Amount of mercury in shoot | **P<0.01** | **P<0.01** | **P<0.01** |
| Amount of mercury in tuber | **P<0.01** | **P<0.01** | **P<0.01** |
| Amount of mercury in root | **P<0.01** | **P<0.01** | **P<0.01** |
| Total amount of mercury | **P<0.01** | **P<0.01** | **P<0.01** |
